# Association of Veggie Meter–Assessed Skin Carotenoids and Dietary Intake Among Indigenous Families: The Indigenous Supported Agriculture “Go Healthy” Study

**DOI:** 10.1016/j.cdnut.2025.107521

**Published:** 2025-08-05

**Authors:** Susan B Sisson, Emma Kasahara, Shanon Casperson, Stephanie Jilcott Pitts, Jessica Reese, Ying Zhang, Tori Taniguchi, Kaylee R Clyma, Jann Hayman, Valarie Blue Bird Jernigan

**Affiliations:** 1 Department of Nutrition Sciences, University of Oklahoma Health Sciences Center, Oklahoma City, Oklahoma, United States; 2 USDA Agricultural Research Services, Grand Forks Human Nutrition Research Center, Grand Forks, North Dakota, United States; 3 Department of Public Health, East Carolina University, Greenville, North Carolina, United States; 4 Department of Biostatistics and Epidemiology, University of Oklahoma Health Sciences Center, Oklahoma City, Oklahoma, United States; 5 Center for Indigenous Health Research and Policy, Oklahoma State University Center for Health Sciences, Tulsa, Oklahoma, United States; 6 Department of Natural Resources, Osage Nation, Pawhuska, Oklahoma, United States

**Keywords:** skin carotenoids, Native American, fruit and vegetables intake, household health behaviors, Veggie Meter, reflection spectroscopy

## Abstract

**Background:**

Indigenous communities have seldom been included in previous research on skin carotenoid scores (SCS) and diet. Further, little is known about familial SCS.

**Objectives:**

This study aimed to examine associations between SCS and body mass index (BMI), dietary intake among adults and children in Osage Nation, and examine associations between SCS among family members.

**Methods:**

This cross-sectional study examined SCS measured using a Veggie Meter. Adults completed a single 24-h diet recall to calculate the Healthy Eating Index (HEI)-2015. Linear mixed modeling was used to examine associations, while accounting for family correlation. Linear regression (adjusting for age, sex, BMI, tobacco use, and season) was used to examine associations between SCS and diet. Spearman correlation were used to examine associations between SCS between household members.

**Results:**

Among the study population, 61% of adults (*n* = 445) was females and 93% overweight/obese; HEI was 42.7 ± 11.2; and SCS was 211.8 ± 57.9. Further, 55.6% of children (*n* = 135, 3–17 y) was females and 45.2% overweight/obese, with HEI of 207.8 ± 62.0. In adults, SCS were higher in males than that in females (226.0 ± 61.0 compared with 203.6 ± 55.1; *P* < 0.001). Children <10 y had higher SCS than those ≥10 y (221.4 ± 65.8 compared with 194.2 ± 55.5; *P* = 0.017). Children with obesity (178.4 ± 44.0) had significantly lower SCS than children in other weight classifications (*P* = 0.023). HEI was significantly associated with SCS (β: 0.50; 95% CI: 0.01, 0.99). Household adult SCS was significantly correlated (*r* = 0.26; *P* = 0.005). SCS among children <10 y were significantly associated with adult SCS (*r* = 0.34; *P* = 0.037). No correlations were observed in SCSs between children ≥10 y and adults.

**Conclusions:**

Adult males, younger (<10 y) children, and nonobese children show higher SCS. A healthier overall diet, as measured by HEI, is associated with higher SCS in adults, while controlling for covariates. Within families, SCS between adults were correlated, indicating similar dietary intake of fruit and vegetables. Adult SCS within households is associated with young children’s (<10 y) SCS, but not with that of older children (≥10 y).

## Introduction

Fruit and vegetable (FV) intake is inversely associated with incidence of many chronic diseases, including diabetes [[1], [2], [3]], certain cancers [[4], [5], [6], [7], [8]], cardiovascular disease [[9], [10], [11]], stroke [12], and obesity [13]. The impact of these diseases is notable with 38.4 million Americans living with diabetes [14], >80,000 new cancer diagnoses per year attributed to diet [15], 1 person dying from cardiovascular disease every second in the United States [16], annual costs of strokes exceeding $56 billion dollars [17], and 40% of the American population living with obesity [18]. Increased FV intake can also decrease risk of all-cause mortality [[19], [20], [21]] and improve mental health [22,23] and happiness [24]. In particular, intake of carotenoid-rich foods has been associated with decreased chronic disease risk [25]. Unfortunately, most Americans do not meet recommendations for FV intake [26]. One way to increase FV intake among children is to intervene at the family level by increasing intake among all household family members. This is because FV intake among children is correlated with parental modeling and intake [27]. Spousal partners also share dietary intake patterns and may provide support for healthy dietary behaviors [28]. For example, 70% of couples are concordant regarding FV intake [29].

Significant research resources are dedicated to investigating the association between FV intake and reduced chronic disease incidence and investigating the effectiveness of FV interventions across the lifespan and within families. This research may be particularly relevant to indigenous communities, because these communities experience disproportionate rates of cardiometabolic disease, including 50% higher risk of obesity, 30% higher risk of hypertension, and twice risk of diabetes, than non-Hispanic White communities [30]. Although limited research has been conducted on the social and cultural context of eating within Native American families, a recent study found that higher social support was related to lower consumption of added sugars, sugar-sweetened beverages, and red/processed meats [31]. More research is needed to confirm the moderating effect of family social support on dietary behaviors, particularly within Native American families.

However, research about FV intake can be difficult due to the challenging nature of assessing dietary intake, which can be affected by recall and desirability biases [32,33], particularly in communities that are further from academic health centers where participation in research may be reduced. Novel technologies are thus needed to augment traditional self-report methods of dietary intake assessment [34]. One such technology is the use of a device called the Veggie Meter, which uses pressure-mediated reflection spectroscopy as an objective, noninvasive, portable, rapid assessment of carotenoids in human skin [35]. Skin carotenoid scores (SCSs) are biomarkers of dietary intake of carotenoid-rich FVs [36], and levels measured by the Veggie Meter in adults highly correlate with total serum carotenoids (*r* = 0.81; *P* < 0.001) [37] and total plasma carotenoids (*r* ≥ 0.70; *P* < 0.001) [38,39]. SCS measured by the Veggie Meter are also correlated with self-reported dietary recalls in adults and children, including the Harvard Semi-Quantitative Food Frequency Questionnaire [39,40], Carotenoid-Specific Food Screener [41,42], National Cancer Institute FV Screener [43], previous day FV intake in elementary children [44], and the Kids Bites Food Frequency Questionnaire in toddlers [45]. The Veggie Meter has been used for SCS assessments in several populations of adults and children, including predominantly White [40,[46], [47], [48], [49]] and racially diverse groups that included Asian, Hispanic, and African American populations [39,41,43,[50], [51], [52]]. Recently, SCS have been inversely associated with cardiovascular disease risk and metabolic syndrome in a Japanese population [53,54].

Indigenous populations historically have not been included in these studies, leading to a lack of information on Veggie Meter skin carotenoid measurements in this population. Further, associations between SCSs and dietary intake of FV within families have not been investigated in any population. This research aimed to focus on describing the association between Veggie Meter–assessed SCS and demographic characteristics and BMI (in kg/m^2^), among adults and children in an indigenous community. In addition, the association of Veggie Meter–assessed SCS and adult’s self-reported dietary intake is described. Lastly, the association of Veggie Meter–assessed SCS within families is determined. We hypothesized that SCS within households will be correlated and that SCS and dietary intake of FVs will be associated.

## Methods

### Study design

This cross-sectional research analyzes baseline data from an RCT known as the Indigenous Supported Agriculture (ISA) “Go Healthy,” study (NCT05773833). The ISA study is a community-based participatory research (CBPR) study in partnership with the Osage Nation in northeastern Oklahoma. Methods from the ISA study relevant to this research are summarized further. Go Healthy sample size was determined for the primary aim of determining intervention effectiveness; baseline data were included in this analysis.

### Community setting

The study uses a CBPR approach, which combines education and social action to reduce health disparities and improve health by working with community members as research partners to match research goals to the community’s goals [55]. As such, Osage Nation members were involved in the research team and participated in all levels of the study design, evaluation and survey development, recruitment, data collection, interpretation, and dissemination. The Osage Nation occupies the only federally recognized reservation in Oklahoma [56]. Approximately half of tribal members live on the reservation, a quarter of the reservation’s residents live in poverty, and more than three-quarters of the tribe’s members are overweight. The Osage Nation runs several programs to support its families, such as a Community Health Representative Program, a Community Health Department, and the Food Distribution Program on Indian Reservations (FDPIR). FDPIR is a program operated by the USDA, which provides monthly canned and packaged foods to tribal members with limited access to the Supplemental Nutrition Assistance Program.

In 2013, the Osage Nation founded Harvest Land Farm, formerly known as Bird Creek Farm, to facilitate indigenous food sovereignty, which refers to the right and responsibility of indigenous people to access healthy and culturally appropriate foods made through traditional indigenous practices [[57], [58], [59]]. Indigenous food sovereignty supports communities by promoting access to healthy and traditional foods and reducing dependence on packaged and processed foods. It also supports public health efforts to improve health outcomes by reducing diet-related health disparities [60]. Harvest Land has since grown and has started providing fresh produce such as tomatoes, strawberries, squash, zucchini, lettuce, cucumbers, corn, variety of peppers, onions, and pineapples to the community. Harvest Land and the Osage Nation have collaborated with this study team on previous research, the National Institute on Minority Health and Health Disparities-funded FRESH farm-to-school program (R01MD011266), also a CBPR study [[61], [62], [63]]. The relationships forged from FRESH are the foundation of this ISA study.

### Recruitment

Variety of recruitment events were held to reduce bias of enrollment. Participants were recruited in the community (e.g., Osage media, town hall meetings, and community events) and in clinic (Osage Health System) settings by Osage staff and community-based study staff. These individuals were then informed of the study goals, study design, and participation requirements so they could decide whether to participate or not. This study was approved by university and tribal institutional review board.

Recruited individuals served as the index participants since multiple people from a single household could enroll. Eligibility criteria for index participants included the following: *1*) being aged 18–75 y; *2*) identifying as Native Americans (NAs); *3*) residing within Osage Nation and not planning to move in the next 2 y; *4*) being classified as overweight or obese (BMI ≥ 25); and *5*) were not pregnant or planning to become pregnant in the next 2 y. It is worthy to note that individuals who identify as NA, but not necessarily Osage, could be eligible to participate as residence in Osage Nation is open to all people. Further, ≤2 additional adults and ≤4 children aged 3–17 y old who lived in the same household as the index participant could also be enrolled. These individuals did not need to be NA or be classified as overweight or obese but must live with the index participant.

### Procedures

All participants completed baseline data collection prior to household-level randomization into the ISA study (February through November of 2023). Several data were collected from each participant for this baseline data collection by trained personnel. The following data collected were relevant to this study: a single 24-h dietary recall, BMI (calculated from weight and height measurements), and skin carotenoids (measured using the Veggie Meter). These data were collected for all adult participants (index participants and additional adults in the same household). For children, only BMI percentile and skin carotenoids were collected.

### Measures

#### Skin carotenoid measurement

Skin carotenoids, the dependent variable, were quantified using the Veggie Meter. The ring finger of the nondominant hand was gently pressed against a convex lens with the help of the spring-loaded lid [37,64]. The pressure technique of the convex glass lens enhances light from the white LED source and reflection optical signals [37,64]. Blood is temporarily pushed away from the fingertip tissue, allowing optical assessment of reflected light detected by the spectrograph [37,64]. The interfaced computer software adjusts for light exposure, residual tissue blood, and melanin content and displays a reflection score ranging from 0 to 800 [37]. Participants’ hands were washed or wiped with alcohol wipes prior to scans. The lens was wiped with alcohol or a microfiber cloth prior to scans and was wiped once for each participant’s 3 scans, rather than once before each scan. A single measure takes only 10 seconds. Triplicate scans were taken to enhance rigor. To reduce reactivity, participants were provided their Veggie Meter score but were not allowed to see the screen during recording [65]. The Veggie Meter device was recalibrated every hour of continuous use following factory recommended procedures [66]. An mean of 3 scans was calculated and used for all analyses. For sensitivity analyses, the average of the closest 2 scans was also calculated and used in analyses. The Veggie Meter was demonstrated at planning events with community members involved on the research team. However, it was not demonstrated with community members during recruitment to minimize reactivity.

#### Dietary intake

Diet intake, the independent variable, was assessed in a single-day 24-h recall using the Automated Self-Administered-24 h Dietary Assessment tool (ASA-24) [67]. Cup equivalents of total fruit, total vegetables, total red and orange vegetables, and total FVs were calculated. This self-report instrument generates data that can be used to calculate the Healthy Eating Index (HEI), with scores ranging from 0 (least healthy) to 100 (healthiest) [68]. Relevant HEI subcomponents (total vegetables, total fruit, greens and beans, and whole grains) were also calculated. HEI version 2015 was used for this study, which has demonstrated sufficient validity and reliability [69]. HEI has been widely used in public health research with diverse populations, including 1 analysis that used the previous version (HEI-2010) to assess diet of the FDPIR recipients administered by the United States federal government [70]. The HEI scores were calculated using publicly available SAS code [71].

ASA-24 was self-directed, and participants were given their login information to complete the recall. If participants had not yet completed the recall after 3 reminder emails, they were contacted via phone call and/or text message and offered the opportunity to complete the recall over the phone instead. Those who completed the recall over the phone did so with a university Dietetics and Nutritional Sciences graduate student. The researcher logged into the ASA-24 website using the participant’s login information and followed the prompts on the screen to input participant information. If participants did not want to complete the recall over the phone, they were emailed their login information again.

#### Covariates

Covariates relevant to this study included age in years, sex (female, male, transgender, Two Spirit, do not identify and female, other, and prefer not to answer), tobacco use (categorized as current, former, and never), BMI (measured by trained community technicians), and month of data collection (recorded by research technician).

#### Analyses

Means, SDs, and frequencies were used to describe the SCS of adults and children. Multivariate mixed linear modeling was used to examine the association of descriptive characteristics and SCS in adults and children. In adults, multivariate mixed linear regression models were used to determine the association of dietary intake and SCS. In the case of significant sex interactions, stratified analyses were calculated. These models accounted for clustering within families and were calculated crude and with all covariates (age, sex, BMI, tobacco use, and month of data collection). Missing data were excluded from a respective analysis. SCS were slightly skewed, thus Spearman correlations were used to examine association between SCS within the same household. Household correlations were computed for 3 stratified groups: *1*) index adult and another adult participant in the household (*n* = 115); *2*) the index adult and a child aged 3–10 y in the household (*n* = 39); and *3*) the index adult and a child aged 11–17 y in the household (*n* = 47). These age groups were stratified due to the separation of childhood and adolescence [72] and the median age in the data set.

## Results

**FIGURE 1:**
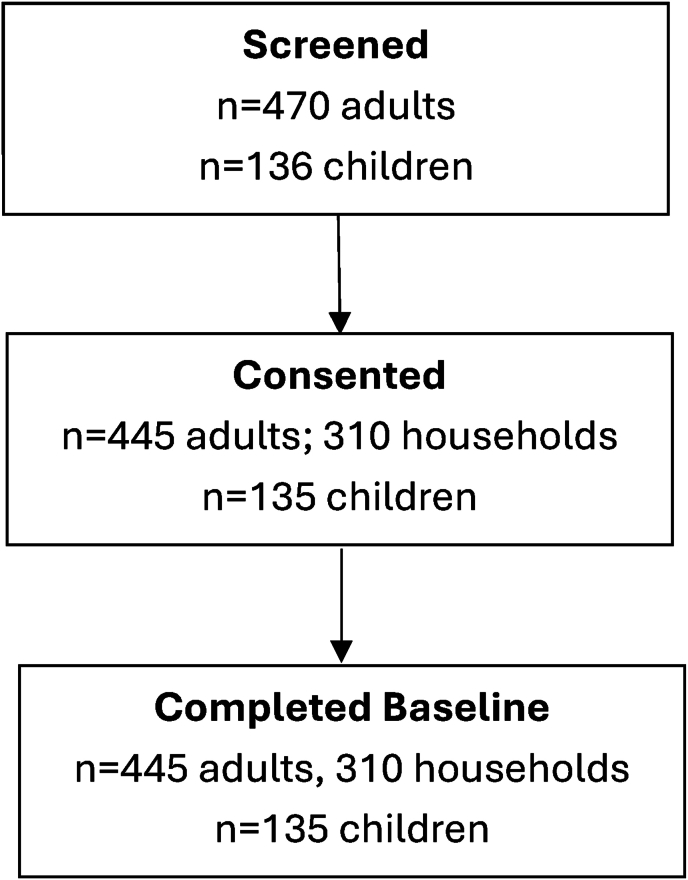
Flow diagram of participant screening, consent, and completion of baseline measures in the Indigenous Supported Agriculture “Go Healthy” trial in Osage Nation 2023.

In total, 470 adults were screened; 445 adults, representing 310 households consented and completed baseline measures; 136 children within those households with an eligible adult were screened; 135 consented and completed baseline measures (Figure 1). Less than 5% of participants had a missing value for an individual variable (TABLE 1, TABLE 2). Adult participants (*n* = 445) were 60.9% females, 93% were overweight or obese, 46.1% currently smoked, and were aged 44.0 ± 16.1 y (Table 1). The mean HEI-2015 for adults was 42.7 ± 11.2. Adult participants self-reported consuming 0.4 ± 0.9 cups/d of fruit, 1.3 ± 1.2 cups/d of vegetables, 0.31 ± 0.5 cups/d of red and orange vegetables, and 1.70 ± 1.5 cups/d total FVs. Children (*n* = 135) were 55.6% female, 45.2% were overweight or obese, and were 10.1 ± 4.3 y (Table 2). Average SCS for adults was 211.8 ± 57.9, and mean SCS for children was 207.8 ± 62. The majority of data collection occurred in the fall season including September, October, and November (53.5%).

**TABLE 1:** Descriptive statistics of adults in the Indigenous Supported Agriculture “Go Healthy” study in Osage Nation 2023.

Variable	Frequency (%)	Mean (SD)
Total number	445	
No. of adults/household		
1	195 (62.9)	
2	95 (30.7)	
3	20 (6.4)	
Age (y)	44.0 (16.1)	
18–24	44 (9.9)	
25–34	75 (16.9)	
35–44	97 (21.8)	
45–54	80 (18.0)	
55–64	73 (16.4)	
65+	61 (13.7)	
Missing	15 (3.3)	
Gender		
Male	156 (35.1)	
Female	271 (60.9)	
Missing	18 (4.0)	
BMI		
Underweight (<18.5)	0 (0)	
Healthy weight (18.5–24.9)	27 (6.1)	
Overweight (25.0–29.9)	121 (27.2)	
Obesity (≥30.0)	293 (65.8)	
Missing	4 (0.9)	
Current smoking		
Yes	205 (46.1)	
No	220 (49.4)	
Missing	20 (4.5)	
Highest level of education		
High school graduate/GED or Less	144 (32.4)	
Some postsecondary	193 (43.4)	
College graduate or more	88 (19.7)	
Missing	20 (4.5)	
ASA-24		
Total fruits (cups/d)		0.41 (0.85)
Total vegetables (cups/d)		1.29 (1.21)
Total red and orange vegetables (cups/d)		0.31 (0.45)
Total fruits and vegetables (cups/d)		1.70 (1.54)
HEI-2015		42.66 (11.23)
HEI component—total vegetables (points)		2.8 (1.9)
HEI component—greens and beans (points)		1.2 (2.0)
HEI component—total fruit (points)		1.0 (1.8)
HEI component—whole fruit (points)		1.0 (1.8)
HEI component—whole grains (points)		1.7 (3.1)
Missing ASA-24	15 (3.4)	
Veggie Meter skin carotenoid score		
First score		205.5 (60.0)
Missing first score	1 (0.2)	
Second score		215.3 (61.5)
Missing second score	1 (0.2)	
Third score		214.5 (63.4)
Missing third score	1 (0.2)	
Average of the 3 scores		211.8 (57.9)
Average of the 2 closest scores		211.3 (59.5)
Missing all scores	1 (0.2)	
Month of skin carotenoid score data collection		
February	7 (1.6)	
March	55 (12.4)	
April	35 (7.9)	
May	50 (11.2)	
June	15 (3.4)	
July	27 (6.0)	
August	17 (3.8)	
September	24 (5.4)	
October	93 (20.9)	
November	121 (27.2)	
Missing	1 (0.2)	
Season of skin carotenoid score data collection		
February–May (Spring)	147 (33.0)	
June–August (Summer)	59 (13.3)	
September–November (Fall)	238 (53.5)	
Missing	1 (0.2)	

Abbreviations: HEI, Healthy Eating Index; ASA, Automated Self-Administered Dietary Assessment.

**TABLE 2:** Descriptive statistics of Indigenous children in the Go Healthy: Indigenous Supported Agriculture study in Osage Nation 2023.

Variable	Frequency (%)	Mean (SD)
Total number	135	
No. of household clusters		
1 Child	23 (34.9)	
2 Children	23 (34.9)	
3 Children	14 (21.2)	
4 Children	6 (9.0)	
Age (y)	10.1 (4.3)	
3	6 (4.5)	
4	6 (4.5)	
5	11 (8.1)	
6	12 (8.9)	
7	11 (8.1)	
8	8 (5.9)	
9	10 (7.4)	
10	11 (8.1)	
11	5 (3.7)	
12	12 (8.9)	
13	6 (4.5)	
14	5 (3.7)	
15	9 (6.7)	
16	14 (10.4)	
17	8 (5.9)	
Missing	1 (0.7)	
Gender		
Male	59 (43.7)	
Female	75 (55.6)	
Missing	1 (0.7)	
BMI percentiles for age and gender		
Underweight (<5%)	3 (2.2)	
Healthy weight (5 to <85%)	71 (52.6)	
Overweight (85 to <95%)	28 (20.7)	
Obesity (≥95%)	33 (24.5)	
Missing	0 (0)	
Veggie Meter–assessed skin carotenoid score (SCS)		
First score		207.4 (64.6)
Missing first score	0 (0)	
Second score		207.9 (64.7)
Missing second score	0 (0)	
Third score		208.2 (63.9)
Missing third score	0 (0)	
Average of the 3 scores		207.8 (62.1)
Average of the 2 closest scores		207.1 (63.6)
Missing all scores	0 (0)	
Month of vegetable meter collection		
June	14 (10.4)	
July	17 (12.6)	
August	15 (11.1)	
September	15 (11.1)	
October	38 (28.1)	
November	32 (23.7)	
Missing	4 (3.0)	
Season of Vegetable Meter collection		
Summer	46 (34.1)	
Fall	85 (62.9)	
Missing	4 (3.0)	

Abbreviation: SCS, skin carotenoid score.

Table 3 includes SCS by demographic characteristics for adults and children. In adults, males had higher SCS than females (226.0 ± 61.0 compared with 203.6 ± 55.1; *P* < 0.001). In children, those younger than 10 y had higher SCS than those older than 10 y (221.4 ± 65.8 compared with 194.2 ± 55.5; *P* = 0.017). In children, there was a difference in SCS across weight classifications (*P* = 0.004). Post hoc analyses revealed that children with obesity (178.4 ± 44.0) had significantly lower SCS than other weight classifications (*P* = 0.023). There were no differences between underweight (273.4 ± 29.8), healthy weight (211.6 ± 64.9), and overweight (226.0 ± 63.1) individuals. There were no correlations between SCS and BMI, age, HEI-2015, or FV intake in adults or between SCS and BMI percentile or age in children (Table 4).

**TABLE 3:** Mean and SD Veggie Meter® skin carotenoid scores according to demographic characteristics among indigenous adults and children.

Variable	Skin carotenoid score (SD)	*P*^1^
Adults (*n* = 445)		
Age (y)		
18–24	201.0 (44.1)	0.051
25–34	211.1 (51.8)	
35–44	211.9 (49.6)	
45–54	207.4 (56.5)	
55–64	203.4 (55.8)	
65+	235.3 (82.8)	
Gender		
Male	226.0 (61.0)	**<0.001**∗
Female	203.6 (55.1)	
BMI		
Healthy weight (18.5–24.9)	209.7 (62.6)	0.479
Overweight (25.0–29.9)	217.4 (59.3)	
Obesity (≥30.0)	210.2 (57.1)	
Current smoking		
Yes	208.4 (62.5)	0.266
No	215.4 (53.6)	
Highest level of education		
High school graduate/GED or less	211.0 (56.7)	0.248
Some postsecondary	207.0 (57.7)	
College graduate or more	222.4 (58.8)	
Season of Vegetable Meter collection		
Spring	220.2 (54.4)	0.051
Summer	217.1 (63.6)	
Fall	205.1 (58.0)	
Children (*n* = 135)		
Age (y)		**0.017**∗
<10 preadolescence	221.4 (65.8)	
≥10 adolescence	194.2 (55.5)	
Gender		
Male	212.9 (64.8)	0.342
Female	202.7 (59.7)	
BMI percentiles for age and gender		
Underweight (<5%)	273.4 (29.8)	**0.004**∗
Healthy Weight (5% to <85%)	211.6 (64.9)	
Overweight (85% to <95%)	226.0 (63.1)	
Obesity (≥95%)	178.4 (44.0)	
Season of Vegetable Meter collection		
Summer	194.5 (62.6)	0.094
Fall	215.4 (62.2)	

1 *P* values are from univariate linear mixed models accounting for the correlation of those living in the same household.

∗ Statiscal significance

**TABLE 4:** Spearman Correlation between average of 3 skin carotenoid scores and BMI, age, cups of fruit and vegetable intake, HEI-2015, and HEI-2015 components in adults, children 10 y and over, and children under 10 y.

Variable	*n*	Spearman correlation coefficient	*P*
Adults (18–75 y)s			
BMI	442	−0.0251	0.5981
Age	430	0.0339	0.4830
Total fruit (cups/d)	430	0.0238	0.6230
Total vegetable (cups/d)	430	0.0704	0.1451
Red/orange vegetable (cups/d)	430	0.0403	0.4051
HEI-2015	430	0.0693	0.1516
HEI component—total vegetables (points)	430	0.1107	**0.0217**
HEI component—greens and beans (points)	430	0.0906	0.0605
HEI component—total fruit (points)	430	0.0252	0.6028
HEI component—whole fruit (points)	430	0.0690	0.1532
HEI component—whole grains (points)	430	0.0252	0.6019
Preadolescence (3–9 y)			
BMI percentile	64	−0.0473	0.7105
Age	64	−0.1273	0.3162
Adolescence (10–17 y)			
BMI percentile	70	−0.1847	0.1259
Age	70	0.1354	0.2638

Abbreviations: HEI, Healthy Eating Index.

In the adjusted models, HEI-2015 total score (Table 5) was significantly associated with SCS (β: 0.50; 95% CI: 0.01, 0.99). HEI-2015 subcomponent total vegetables (β: 3.30; 95% CI: 0.30, 6.26) and greens and beans (β: 3.12; 95% CI: 0.30, 5.93) were associated with SCS. Sex-stratified, adjusted models indicate that in females only, HEI-2015 subcomponent whole fruit was significantly associated with SCS (Table 6) (β: 3.75; 95% CI: 0.28, 7.23). No association was observed in males. Self-reported cups/day of fruit, vegetables, red and orange vegetables, total FVs, HEI-2015 subcomponent total fruit, and HEI-2015 subcomponent whole grains were not associated with SCS.

**TABLE 5:** Univariate and multivariable analysis for the association between self-reported dietary intake of fruits, vegetables, and the 2015 Healthy Eating Index and skin carotenoid scores as determined by the Veggie Meter among Indigenous adults.

Main predictor	Estimate (95% CI)^1^		
	Univariate	Adjusted^2^	Reduced^3^
Total fruit consumption (cups/d)	0.50 (−5.90, 6.90)	0.22 (−6.25, 6.68)	0.56 (−5.81, 6.93)
Total vegetable consumption (cups/d)	2.98 (−1.62, 7.58)	1.68 (−2.97, 6.32)	2.28 (−2.30, 6.86)
Total red and orange vegetable consumption (cups/d)	3.94 (−8.35, 16.22)	3.42 (−8.92, 15.76)	2.52 (−9.72, 14.76)
Total fruit and vegetable consumption (cups/d)	2.01 (−1.61, 5.62)	1.11 (−2.55, 4.78)	1.59 (−2.01, 5.19)
Healthy Eating Index—2015	0.43 (−0.06, 0.92)	0.30 (−0.21, 0.81)	**0.50 (0.01, 0.99)**
HEI component—total vegetables (points)	**3.50 (0.52, 6.49)**	**3.13 (0.08, 6.17)**	**3.30 (0.30, 6.26)**
HEI component—greens and beans (points)	**3.00 (0.20, 5.79)**	**2.91 (0.01, 5.81)**	**3.12 (0.30, 5.93)**
HEI component—total fruit (points)	0.66 (−2.41, 3.72)	1.12 (−2.03, 4.27)	1.47 (−1.62, 4.54)
HEI component—whole grains (points)	0.09 (−1.64, 1.83)	0.07 (−1.71, 1.85)	0.39 (−1.33, 2.11)

Abbreviation: HEI, Healthy Eating Index.

1 From linear mixed models accounting for the correlation of those living in the same household.

2 Adjusted for age, gender, BMI, current smoking, and season of skin carotenoid score collection.

3 Used manual backward selection to reduce the model. At an α of 0.5 only gender remained in the reduced models, except when the main predictor was total red and orange vegetable consumption or the HEI components for greens/beans and total fruit, where gender and season remained in the model. Used crossproduct terms to evaluate 2-way effect modification between the main predictor and the covariates that remained in the reduced model. At an α of 0.05, a 2-way effect modification was observed.

**TABLE 6:** Univariate and multivariable analysis stratified by sex for the association 2015 Healthy Eating Index subcomponent while fruit and skin carotenoid scores as determined by the Veggie Meter among Indigenous adults.

Main predictor	Estimate (95% CI)^1^		
	Univariate	Adjusted^2^	Reduced^3^
HEI component—whole fruit (points) for females	**3.93 (0.01, 6.99)**	2.91 (−0.86, 6.68)	**3.75 (0.28, 7.23)**
HEI component—whole fruit (points) for males	−4.51 (−11.36, 2.34)	−4.99 (−15.20, 5.21)	−4.51 (−11.36, 2.34)

Abbreviation: HEI, Healthy Eating Index.

1 From linear mixed models accounting for the correlation of those living in the same household.

2 Adjusted for age, gender, BMI, current smoking, and season of skin carotenoid score collection.

3 Used manual backward selection to reduce the model. At an alpha of 0.5 only season remained in the reduced models for females and no variables remained in the reduced models for males.

**FIGURE 2:**
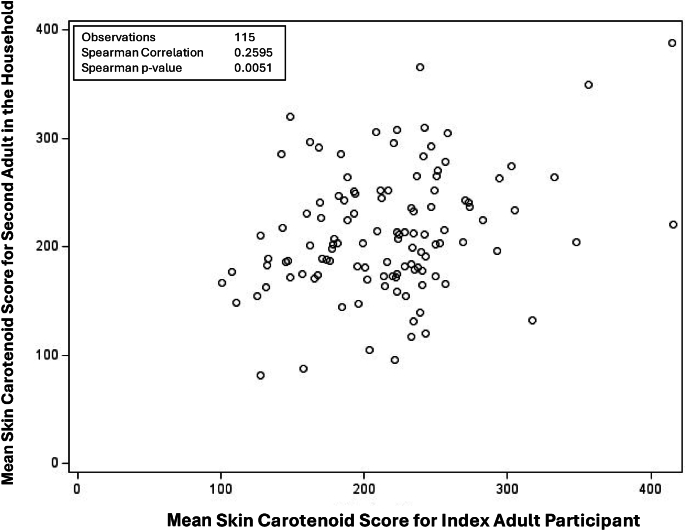
Scatter plot for the correlation between average skin carotenoid scores for the index adult participant and additional adult participant from the same household among indigenous families in 2023.

**FIGURE 3:**
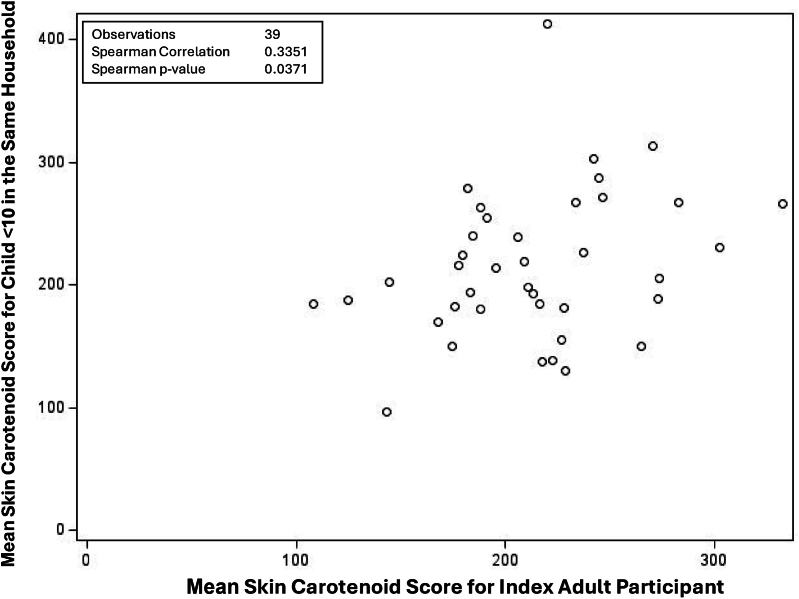
Scatter plot for the correlation between average skin carotenoid scores for the index adult participant and a child participant aged <10 y from the same household among indigenous families in 2023.

**FIGURE 4:**
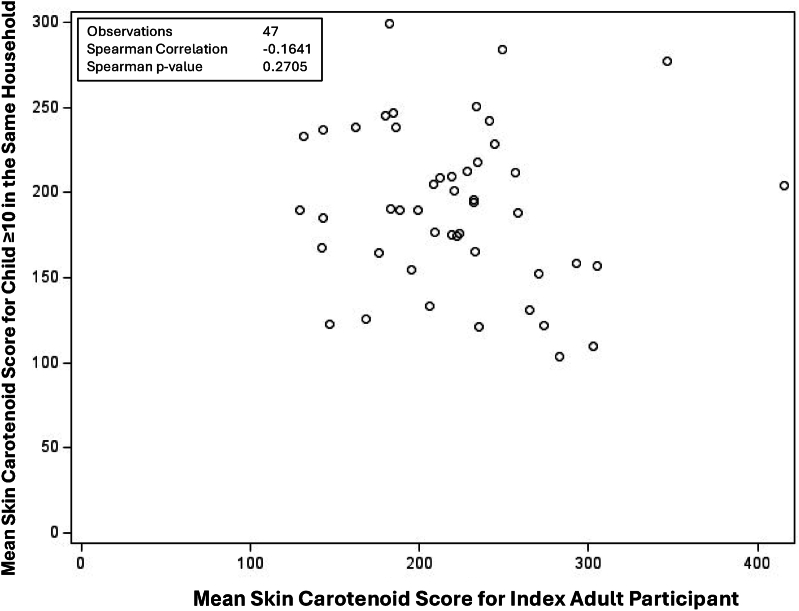
Scatter plot for the correlation between average skin carotenoid scores for the index adult participant and a child participant aged ≥10 y from the same household among indigenous families.

There was a significant correlation (*r* = 0.26, *P* = 0.005) between the index adult’s SCS and the SCS of the additional adult (*n* = 115) in the household (Figure 2). In children younger than 10 y of age (*n* = 39) (Figure 3), there was a significant correlation between the index adult and the child’s SCS (*r* = 0.34; *P* = 0.037). In children 10 y and older (*n* = 47) (Figure 4), there was no significant correlation between SCSs of index adults and those of children.

## Discussion

The primary findings from this study demonstrated that healthier dietary patterns, as determined by overall HEI-2015 and subcomponent scores, were associated with higher SCS in adults. To our knowledge, this study is the first to demonstrate this finding in an indigenous population. However, SCSs were not associated with self-reported dietary intake of fruit, vegetables, or red/orange vegetables. SCSs in this study were 211.8 in adults and 207.8 in children, which are slightly lower than SCSs of Alaska Natives (ANs) in a previous study (220.1) [73]. HEI in this study (42.6) was lower than the national average of 56.6 [69] and a sample of NA youth (61.2) [74], but closer to another study of NAs in the Midwest and Southwest (49.3) [74]. The total FV consumed in this study was 1.70 cups/d (0.41 cups/d of fruit and 1.29 cups/d of vegetables), lower than the 2.5 servings/d reported in ANs [73].

Studies using a 24-h recall, such as the ASA-24, have demonstrated no [38,73] or weaker associations [38,73]. Although Hill et al. [73] observed a difference in SCS among ANs who consumed ≥1 serving of FV/d compared with those who consumed none per day, a continuous analysis of FV intake using the ASA-24 was not associated with SCS. Our findings of no observed association between SCS and FV intake, as measured by the ASA-24, add to the literature. However, SCS has been associated with FV intake as reported by food frequency questionnaires [39,[41], [42], [43],^51^,^75^,^76^]. These differences in SCS associated with self-reported FV intake is perhaps due to recall bias and the timeframe of a single 24-h recall compared with the multiweek assessment of SCS by the Veggie Meter. Additional explanation could also be that FV consumed in this sample does not include substantial carotenoid-rich varieties. Orange and red vegetables constituted only 24% of the total vegetable consumption in this sample. Although our findings were null, in regards to SCS and FV intake, there is value in further exploration around these measures and the importance of noninvasive biological measures to complement traditional self-reported assessments.

It is noteworthy to report the significant associations of SCS of adults in the same household as well as between children under 10 y of age and the adults in the household. There was no association of SCS between adults and children 10 y and older in the same household. These findings contribute to the literature by supporting the importance of adult role modeling and children’s dietary intake, especially in young children [27]. Further, it supports the role of familial and spousal support in healthy dietary habits, a relationship that is not clearly defined in the literature [28]. Kurotani et al. [77] recently reported a significant correlation between SCS of parents and children aged 6–15 y in Japan, although this relationship was no longer significant once adjusted for parental smoking and BMI. Pratt et al. [78] reported a significant correlation between SCS of parents and children with a mean age of 12 y. Multiple adults in the household were not examined in either study.

This study did not find associations with BMI and smoking status and SCS; thus, these possible covariates were not adjusted for in our analyses. Further, the age range in this study was broader and stratified analyses between older and younger children. Shared family meals offer a time for connection to community and family as well and provide the opportunity for shared dietary patterns and habits.

Demographic differences in SCS were also observed. Specifically, adult males had higher SCS than adult females. Children under 10 y of age had higher SCS than children who were 10 y and older. Children with obesity had lower SCS than children classified as overweight, healthy weight, and underweight. The differences found in this study are different in comparison with previously published literature [79], namely age, BMI, smoking, and season of data collection were not associated with SCS in adults, and gender and season of data collection were not associated with SCS in children.

Age, gender, body mass, smoking status, and current smoking are among those most examined, predicted to be most influential, and commonly reported covariates in the association of dietary intake and SCS [79]. In adults, age was not associated with SCS in this study, similar to 70% of the studies included in the study by Madore et al. [79] and the single other published study examining SCS in an indigenous population of NAs [73]. Three studies reported that older age was associated with higher SCS, although each of these were conducted outside the United States [40,54,80]. However, in this study, children younger than 10 y of age had higher SCS than children 10 y and older. Previous studies demonstrated mixed findings, although they included a variety of age groups and mostly infants and young children [79,81]. One other study included children across the lifespan [82] and reported that there was no relationship between age and SCS, but this may have been due to the small sample size (*n* = 45).

Adult males had higher SCS than females in this study (226.0 compared with 203.6), which being in agreement with the only other study examining SCS in indigenous populations [73]. Of the 10 studies included in the review by Madore et al. [79], 4 reported no association with sex and 4 reported that females had higher SCS than males; each of those 4 studies were conducted in Asian countries. In this study, there was no difference in SCS in children of different biological sexes. Previous literature has been mixed, with a slight majority of studies showing no association between SCS and biological sex in children [79,81]. In the studies that did demonstrate a difference in SCS by sex, males had higher SCS than females.

In contrast to the majority of previously published studies in adults [79], BMI was not associated with SCS in this study. However, there was very limited variability in BMI in our study sample. An inclusion criterion for the primary intervention was the index adult having a BMI of ≥25.0. Although some adults were not overweight or obese (6.1%), these were the additional adults in the household. Our study demonstrated that children with obesity had lower SCS than children in other weight classifications. A recent review of SCS predictors in children by Hasnin et al. [81] showed that 2 of the 5 studies similarly demonstrated an inverse relationship between BMI and SCS, whereas the other 3 studies showed no relationship.

Smoking status was not associated with SCS in this study, in support of the study by Hill et al. [73]. Further, smoking prevalence in this study was 46%, similar to the reported 40% smokers in the study by Hill et al. [73]. In contrast, most previous studies report smoking as being inversely associated with SCS [79]. Season of data collection was not associated with SCS in this study. One study has demonstrated that SCS were higher in the fall than the winter [83], although other studies included in the review [81] did not demonstrate a difference. Season of data collection is postulated to impact SCS based on the notion that skin carotenoids are lower after more intense UV light exposure [79]. However, season was not associated with SCS as measured by Veggie Meter in this study.

It is prudent to interpret these findings in light of the study’s strengths and limitations. These data were collected using standardized and recommended procedures for assessment of SCS [66] in collaboration with Osage Nation. The large sample size of adults within the same household and children allows for examination of familial relationships in diet previously unexplored in this population. Limitations include the limited variability in BMI of adult participants, precluding the ability to understand the relationship of BMI and SCS in this population. A single 24-h recall, rather than multiple recalls, may have limited the ability to ascertain dietary patterns that may be better associated with long-term intake of carotenoid-rich foods. A dietary carotenoid screener [41] could have additionally been used; however, as this was not the primary study’s purpose and due to participant burden concerns, this was not included. Further, the lack of measures of dietary intake in children limited our ability to further examine the relationships of dietary intake and SCS. The primary study from which these data come was a larger clinical trial focusing on adults, and thus, the participant burden of additional dietary assessment for children was not included. Caution is warranted in generalizing these finds to other populations, and additional research is needed in indigenous populations regarding the validity and reliability of SCS. There are continued opportunities for future research in the assessment of SCS and demographic characteristics, as well as relationship of dietary congruence within households and relationships of SCS with clinical health indices.

In conclusion, this study demonstrated that healthier dietary patterns, as determined by HEI-2015, were associated with higher SCS among adults in Osage Nation. However, SCSs were not associated with self-reported dietary intake of fruit, vegetables, or red and orange vegetables. SCSs of adults living in the same household were weakly positively correlated, as were the SCSs of children under 10 y of age; however, this association was not observed in older children (children 10 y or older), who may be more autonomous in meal choices.

## Author contributions

The authors’ responsibilities were as follows – SBS: designed this analysis plan, research questions, and lead the writing; EK: contributed to literature review; SJP, SC: contributed to relevant literature discussion, interpretation of data, and contributed to analyses, and writing; YZ, JR: are study epidemiologists and statisticians and conducted all analyses, discussed data interpretation, and reviewed all results and interpretations; TT, KC: contributed to ISA study design and coordination of all data collection; VBBJ: is the ISA study Principal Investigator and contributed to all aspects of study design, interpretation, and writing and had final approval of all content; and all authors: have read and approve the final manuscript.

## Data availability statement

Data described in the manuscript, code book, and analytic code may be made available by request and approval of the Osage Nation research review committee.

## Funding

This study was supported by the National Institute on Minority Health and Health Disparities of the National Institutes of Health (R01MD016191). The content is solely the responsibility of the authors and does not necessarily represent the official views of the funders.

## Conflict of interest

VBBJ reports financial support was provided by National Institutes of Health. The other authors report no conflicts of interest.
